# Predicting temporomandibular disorders in adults using interpretable machine learning methods: a model development and validation study

**DOI:** 10.3389/fbioe.2024.1459903

**Published:** 2024-11-05

**Authors:** Yuchen Cui, Fujia Kang, Xinpeng Li, Xinning Shi, Han Zhang, Xianchun Zhu

**Affiliations:** Department of Orthodontic, Hospital of Stomatology, Jilin University, Changchun, Jilin Province, China

**Keywords:** temporomandibular disorders, machine learning, prediction model, shapley additive explanations, random forest

## Abstract

**Introduction:**

Temporomandibular disorders (TMD) have a high prevalence and complex etiology. The purpose of this study was to apply a machine learning (ML) approach to identify risk factors for the occurrence of TMD in adults and to develop and validate an interpretable predictive model for the risk of TMD in adults.

**Methods:**

A total of 949 adults who underwent oral examinations were enrolled in our study. 5 different ML algorithms were used for model development and comparison, and feature selection was performed by feature importance ranking and feature decreasing methods. Several evaluation indexes, including the area under the receiver-operating-characteristic curve (AUC), were used to compare the predictive performance. The precision-recall curve (PR), calibration curve, and decision curve analysis (DCA) further assessed the accuracy and clinical utility of the model.

**Results:**

The performance of the random forest (RF) model was the best among the 5 ML models. An interpretable RF model was developed with 7 features (gender, malocclusion, unilateral chewing, chewing hard substances, grinding teeth, clenching teeth, and anxiety). The AUCs of the final model on the training set, internal validation set, and external test set were 0.892, 0.854, and 0.857, respectively. Calibration and DCA curves showed high accuracy and clinical applicability of the model.

**Discussion:**

An efficient and interpretable TMD risk prediction model for adults was successfully developed using the ML method. The model not only has good predictive performance, but also enhances the clinical application value of the model through the SHAP method. This model can provide clinicians with a practical and efficient TMD risk assessment tool that can help them better predict and assess TMD risk in adults, supporting more efficient disease management and targeted medical interventions.

## 1 Introduction

Temporomandibular disorders (TMD) is a collective term for skeletal and neuromuscular diseases involving the temporomandibular joint (TMJ), masticatory muscles, and associated tissues (Adèrn et al., 2014). It is one of the most common disorders among oral and maxillofacial diseases globally. Clinical manifestations include localized pain in the face and preauricular region, restricted mandibular movement, joint popping and murmurs (Sousa et al., 2019; Yap et al., 2022b; Zhang et al., 2023). Additionally, symptoms such as head and neck pain, dizziness, hearing loss, and earache or tinnitus have been reported (Porto De Toledo et al., 2017; Song et al., 2018; Kang, 2020; 2021; Naderi et al., 2023). Studies have shown that TMD has a high prevalence in adults, with approximately 40%–70% of adults exhibiting at least one sign of TMD (Suzuki and Iwata, 2016), and the prevalence is significantly higher in women than in men (Winocur et al., 2006; Bueno et al., 2018). TMD has a significant impact on the quality of life and oral health of adults. Pain and functional limitations cause patients to suffer from distress in daily activities, including difficulty eating, speaking, and chewing, and psychological problems such as anxiety and depression. In addition, TMD may be associated with sleep disturbances, further affecting patients’ quality of life (Almoznino et al., 2016).

The pathogenesis of TMD is complex and not yet fully defined, and the commonly accepted etiologic theory is the biopsychosocial model, which considers the influence of biological and psychosocial factors (Suvinen et al., 2005; Ohrbach and Dworkin, 2016). Several studies have shown that the etiology of TMD involves anatomical structures, biomechanical factors, psychosocial factors, genetic factors, and environmental factors (Marpaung et al., 2018; Sousa et al., 2019; Zhang et al., 2023), and that the combined effect of these factors contributes to the development and progression of TMD. Although there has been some progress in the research on TMD, there have been few studies on the prediction of TMD risk. Currently, existing risk prediction tools often only cover certain aspects of risk factors and fail to comprehensively and deeply integrate more dimensions of potential risk. Therefore, it is of great significance to comprehensively consider various potential factors leading to TMD and establish a risk prediction model for adult TMD.

With the widespread use of ML in clinical medicine, some ML techniques have been used to develop predictive models for diseases (Lee et al., 2021; Jp et al., 2023). Compared to traditional methods, ML has advantages in handling large-scale data with multidimensional features, accurately identifying disease risk factors, and effectively generating predictive models. Lundberg et al. (2020) proposed the SHAP algorithm, which quantifies the impact of variables on the model through SHAP values, effectively solves the “black box” problem of the ML model that is difficult to interpret, and enhances the transparency and reliability of clinical applications. This study aims to apply ML methods to identify more comprehensively the primary risk factors affecting the occurrence of TMD in adults, develop and validate an interpretable ML risk prediction model, achieve early prediction of TMD, and provide effective auxiliary tools for clinical diagnosis and treatment.

## 2 Materials and methods

### 2.1 Study population

Adults who underwent oral examination at Stomatology Hospital of Jilin University from February 2023 to April 2024 were selected as the study population. Inclusion criteria: adults aged ≥18 years who agreed to participate in the study. Exclusion criteria: (1) systemic diseases; (2) tumours, craniofacial deformities, and craniofacial trauma; (3) undergoing treatment with medications that could mask symptoms of TMD, such as non-steroidal anti-inflammatory drugs or analgesics; and (4) individuals with a history of temporomandibular joint trauma or surgery. The study was approved by the Ethics Committee of the Stomatology Hospital of Jilin University (Approval number: JDKQ2023098) and was conducted under the Declaration of Helsinki. All subjects were informed and consented to participate in the study.

### 2.2 Assessment of TMD

This study used the presence or absence of TMD in adults as the outcome variable. According to the Diagnostic Criteria for Temporomandibular Disorders (DC/TMD) published by the International Society for Dental Research in 2014 (Ohrbach and Dworkin, 2016), subjects who met one or more of the subcategories of the DC/TMD criteria were classified into the TMD group, and those who did not meet these criteria were classified into the no TMD group. The diagnostic criteria for DC/TMD is shown in the Supplementary DC/TMD Diagnostic Criteria.

### 2.3 Data collection

Clinical data were collected from all subjects, including demographic information, oral-related medical history, occlusal factors, oral behavioral habits, lifestyle habits, sleep status, and psychological state in multiple dimensions. These indicators include age, gender, orthodontics, root canal therapy, facial cold stimulation, unilateral chewing, chewing hard substances, chewing gum, biting of soft tissues (lips, tongue, cheeks), grinding teeth, clenching teeth, excessive mouth opening, mouth breathing, uneven or crowded teeth, missing posterior teeth, malocclusion, faulty restoration, prone or lateral sleeping, infrequent exercise, resting chin on the hand, staying up late, prolonged mobile phone use, insomnia, smoking, drinking, obesity, stress, anxiety, and depression, for a total of 29 indicators.

The Generalized Anxiety Disorder 7-item scale (GAD-7) and the Patient Health Questionnaire 9-item scale (PHQ-9) were used to assess patients’ anxiety and depression. The GAD-7 contains 7 items, each item is rated on a 4-point scale from 0 to 3, and the total score ranges from 0 to 21. Rating scale: no anxiety (0-4 points), mild anxiety (5-9 points), moderate anxiety (10-14 points), and severe anxiety (15-21 points). PHQ-9 contains 9 items, each item is rated on a 4-point scale from 0 to 3, with a total score ranging from 0 to 27. Rating scale: no depression (0-4 points), mild depression (5-9 points), moderate depression (10-14 points), moderately severe depression (15-19 points), and severe depression (20-27 points). The Chinese versions of the GAD-7 and the PHQ-9 have been widely used in healthcare organizations and have good reliability and validity (Li et al., 2014).

### 2.4 Model development and evaluation

The development cohort consisted of adults who underwent oral examinations between February 2023 and December 2023 at the Stomatology Hospital of Jilin University, and the external test cohort consisted of adults who underwent oral examinations between February 2024 and April 2024 at the same hospital. This study included 29 predictors. Due to potential multicollinearity among predictors that could affect prediction accuracy, Spearman correlation analysis was used to exclude highly correlated predictors. A heatmap visualization was employed, and among highly correlated features (ρ > 0.7), only one was retained for model construction. The model was developed using predictors that were not highly correlated.

Five different ML algorithms were used for model development and comparison to determine the optimal model. These algorithms included random forest (RF), extreme gradient boosting (XGboost), logistic regression (LR), decision tree (DT), and gradient boosting decision tree (GBDT). To optimize the models, grid search combined with manual tuning was used to obtain the final hyperparameters for each model. The SHAP algorithm was used for feature selection and model interpretation. Initially, the SHAP values of each feature were computed to quantify their contributions to the model’s predictions, and a SHAP summary plot was generated to visualize feature importance. Based on the importance ranking of features, unimportant features were systematically removed while monitoring changes in model performance, aiming to balance performance with complexity. Ultimately, key features were retained, and the model that maintained high predictive performance while being simplified was selected as the final model.

To enhance the model’s reliability, 10-fold cross-validation was applied to the training cohort. In this process, the development cohort was randomly divided into 10 groups. In each iteration, 9 groups were used as the training set to train the model, and the remaining 1 group served as the internal validation set to evaluate the model’s performance on unseen data. Additionally, to further assess the model’s generalizability, an independent external test set was used for evaluation, with data from the test set not involved in the model training process.

The evaluation indexes for the model included AUC, accuracy, sensitivity, specificity, positive predictive value (PPV), negative predictive value (NPV), and F1 score. Furthermore, the precision-recall curve (PR) and area under the PR curve (AP) were used to assess the model’s discrimination, the calibration curves were used to assess the agreement between the predicted and actual probabilities of the model, the Brier scores were used to assess the accuracy of the model, and the decision curves analysis (DCA) were used to assess the clinical utility of the models.

### 2.5 Model explanation

The SHAP method was used to explain the model at both global and local levels. The global explanation demonstrates the relative contribution of each feature to TMD risk and its importance ranking, while the local explanation is specific to a single sample and demonstrates the specific contribution of each feature to the prediction of that sample.

### 2.6 Statistical analysis

Statistical analyses were performed using R version 4.2.3 and Python version 3.11.4. Categorical variables were presented as n (%) and compared using the chi-square test or Fisher’s exact test. For normally distributed continuous variables, they were presented as mean ± standard deviation and compared using the t-test. Continuous variables that did not fit a normal distribution were presented as the median and interquartile range (IQR) and compared using the Mann-Whitney U test. A two-tailed *p*-value < 0.05 was considered statistically significant.

## 3 Results

### 3.1 Baseline characteristics

**FIGURE 1:**
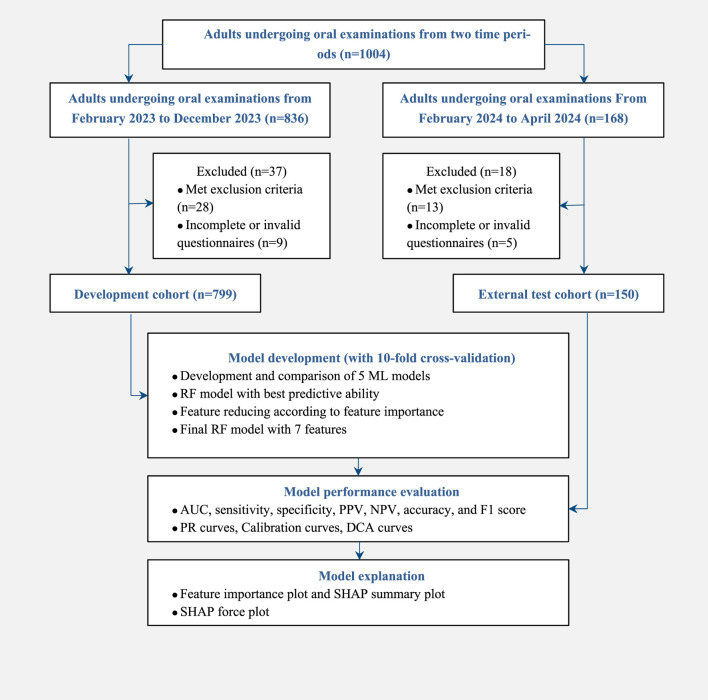
Flow chart of the study design.

From February 2023 to December 2023, 799 adults who underwent oral examinations were included in the development cohort with 460 (57.57%) females and 339 (42.43%) males, and an average age of 36 years. There were 336 (42.05%) subjects with TMD and 463 (57.95%) without TMD. From February 2024 to April 2024, 150 adults who underwent oral examinations were included in the external test cohort, with 92 (61.33%) females and 58 (38.67%) males, and an average age of 35 years. There were 73 (48.67%) subjects with TMD and 77 (51.33%) without TMD. The study flow is shown in Figure 1.

Demographic and clinical characteristics of the development cohort are shown in Table 1, and those of the external test cohort are shown in Supplementary Table S1. Baseline characteristics showed no significant differences (*p* > 0.05) between the development and external test cohorts, indicating a balanced distribution between the two cohorts, as shown in Supplementary Table S2.

**TABLE 1:** Demographic and clinical characteristics on the development cohort.

Characteristic		Total (n = 799)	Non-TMD (n = 463)	TMD (n = 336)	*p*-value
Age, Mean ± SD		36.06 ± 13.22	34.94 ± 13.19	37.59 ± 13.13	0.005
Gender, n (%)	Female	460 (57.57)	229 (49.46)	231 (68.75)	<0.001
	Male	339 (42.43)	234 (50.54)	105 (31.25)	
Orthodontics, n (%)	No	676 (84.61)	404 (87.26)	272 (80.95)	0.015
	Yes	123 (15.39)	59 (12.74)	64 (19.05)	
Root canal therapy, n (%)	No	577 (72.22)	362 (78.19)	215 (63.99)	<0.001
	Yes	222 (27.78)	101 (21.81)	121 (36.01)	
Facial cold stimulation, n (%)	No	716 (89.61)	445 (96.11)	271 (80.65)	<0.001
	Yes	83 (10.39)	18 (3.89)	65 (19.35)	
Unilateral chewing, n (%)	No	411 (51.44)	291 (62.85)	120 (35.71)	<0.001
	Yes	388 (48.56)	172 (37.15)	216 (64.29	
Chewing hard substances, n (%)	No	514 (64.33)	345 (74.51)	169 (50.30)	<0.001
	Yes	285 (35.67)	118 (25.49)	167 (49.70)	
Chewing gum, n (%)	No	686 (85.86)	419 (90.50)	267 (79.46)	<0.001
	Yes	113 (14.14)	44 (9.50)	69 (20.54)	
Biting of soft tissues, n (%)	No	584 (73.09)	376 (81.21)	208 (61.90)	<0.001
	Yes	215 (26.91)	87 (18.79)	128 (38.10	
Grinding teeth, n (%)	No	677 (84.73)	437 (94.38)	240 (71.43)	<0.001
	Yes	122 (15.27)	26 (5.62)	96 (28.57)	
Clenching teeth, n (%)	No	613 (76.72)	405 (87.47)	208 (61.90)	<0.001
	Yes	186 (23.28)	58 (12.53)	128 (38.10)	
Excessive mouth opening, n (%)	No	534 (66.83)	339 (73.22)	195 (58.04)	<0.001
	Yes	265 (33.17)	124 (26.78)	141 (41.96)	
Mouth breathing, n (%)	No	515 (64.46)	325 (70.19)	190 (56.55)	<0.001
	Yes	284 (35.54)	138 (29.81)	146 (43.45)	
Uneven or crowded teeth, n (%)	No	487 (60.95)	316 (68.25)	171 (50.89)	<0.001
	Yes	312 (39.05)	147 (31.75)	165 (49.11)	
Missing posterior teeth, n (%)	No	729 (91.24)	438 (94.60)	291 (86.61)	<0.001
	Yes	70 (8.76)	25 (5.40)	45 (13.39)	
Malocclusion, n (%)	No	505 (63.20)	349 (75.38)	156 (46.43)	<0.001
	Yes	294 (36.80)	114 (24.62)	180 (53.57)	
Faulty restoration, n (%)	No	647 (80.98)	399 (86.18)	248 (73.81)	<0.001
	Yes	152 (19.02)	64 (13.82)	88 (26.19)	
Prone or lateral sleeping, n (%)	No	158 (19.78)	120 (25.92)	38 (11.31)	<0.001
	Yes	641 (80.22)	343 (74.08)	298 (88.69)	
Infrequent exercise, n (%)	No	459 (57.45)	304 (65.66)	155 (46.13)	<0.001
	Yes	340 (42.55)	159 (34.34)	181 (53.87)	
Resting chin on the hand, n (%)	No	456 (57.07)	302 (65.23)	154 (45.83)	<0.001
	Yes	343 (42.93)	161 (34.77)	182 (54.17)	
Staying up late, n (%)	No	259 (32.42)	191 (41.25)	68 (20.24)	<0.001
	Yes	540 (67.58)	272 (58.75)	268 (79.76)	
Prolonged mobile phone use, n (%)	No	234 (29.29)	141 (30.45)	93 (27.68)	0.395
	Yes	565 (70.71)	322 (69.55)	243 (72.32)	
Insomnia, n (%)	No	640 (80.10)	410 (88.55)	230 (68.45)	<0.001
	Yes	159 (19.90)	53 (11.45)	106 (31.55)	
Smoking, n (%)	No	652 (81.60)	383 (82.72)	269 (80.06)	0.338
	Yes	147 (18.40)	80 (17.28)	67 (19.94)	
Drinking, n (%)	No	724 (90.61)	423 (91.36)	301 (89.58)	0.395
	Yes	75 (9.39)	40 (8.64)	35 (10.42)	
Obesity, n (%)	No	684 (85.61)	406 (87.69)	278 (82.74)	0.049
	Yes	115 (14.39)	57 (12.31)	58 (17.26)	
Stress, n (%)	No	457 (57.20)	309 (66.74)	148 (44.05)	<0.001
	Yes	342 (42.80)	154 (33.26)	188 (55.95)	
Anxiety, median [IQR]		4.00 [0.00,7.00]	1.00 [0.00,5.00]	6.00 [4.00,7.00]	<0.001
Depression, median [IQR]		3.00 [0.00,8.00]	1.00 [0.00,5.00]	6.00 [3.00,9.00]	<0.001

Note: Chewing gum (more than 3 pieces per day), Infrequent exercise (less than 75 min of moderate-intensity exercise per week), Prolonged mobile phone use (more than 4 h of use per day), Staying up late (going to bed after 11 p.m. more than 3 nights per week), Insomnia (more than three nights per week), Smoking (1 or more cigarettes per day), Drinking (alcohol consumption for 3 or more days per week), Obesity (BMI > 24).

### 3.2 Model development and performance comparison

Because multicollinearity between features may affect the predictive accuracy, we performed Spearman correlation analysis during model development. The results showed a high correlation between anxiety and depression with a correlation coefficient of 0.83, as shown in Supplementary Figure S1. Based on clinical experience and relevant research, we excluded the feature of depression and used the remaining 28 features for model development.

**FIGURE 2:**
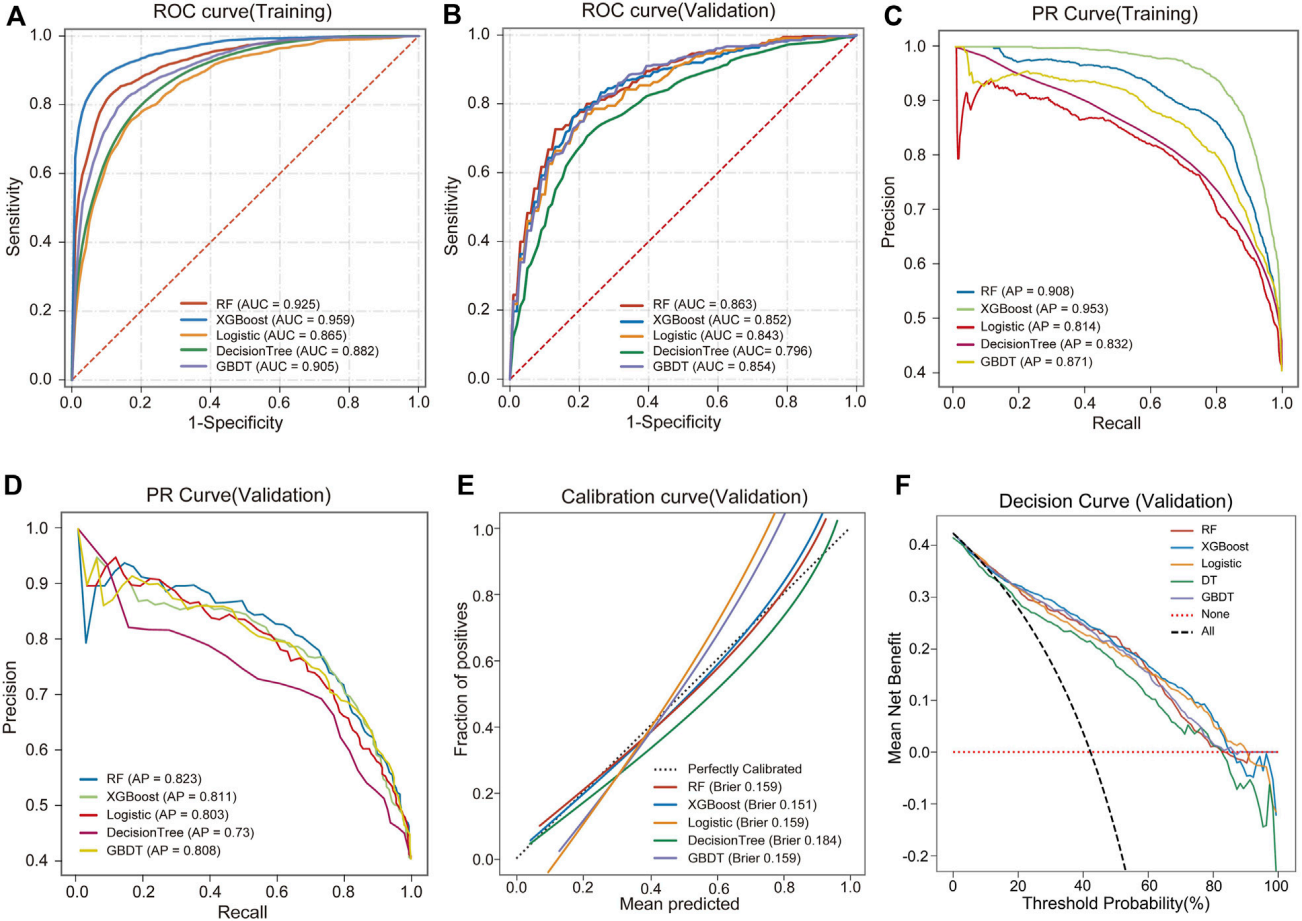
Performance comparison of 5 ML models. **(A)** ROC and AUC of the training set. **(B)** ROC and AUC of the validation set. **(C)** PR curve and AP of the training set. **(D)** PR curve and AP of the validation set. The *y*-axis is precision, and the *x*-axis is recall. The higher the AP value, the better the model performance. **(E)** Calibration curves of the validation set. The *x*-axis is the average prediction probability, the *y*-axis is the actual probability of the event, and the dashed diagonal is the reference line. The closer the fitting line is to the reference line, the lower the Brier score, and the more accurate the model prediction is. **(F)** DCA curves of the validation set. The black dotted line represents the assumption that all patients have TMD, and the red dotted line represents the assumption that no patient has TMD.

We used 5 different ML algorithms to construct a TMD risk prediction model, trained the model through 10-fold cross-validation, and evaluated its performance. The discriminative performance of the 5 models is shown in Table 2. The AUCs of the RF model on the training set and internal validation set were 0.925 and 0.863, respectively, showing the best predictive performance, as shown in Figures 2A, B. The PR curves showed that the average precision (AP) of the RF model on the training set and the internal validation set were 0.908 and 0.823, respectively, and also showed the best discriminative performance, as shown in Figures 2C, D. To comprehensively evaluate the model performance, we also analyzed the calibration curve and the DCA curve. The calibration curve showed that the predicted probabilities of the RF model had good agreement with the actual observations, with a Brier score of 0.159, as shown in Figure 2E. The DCA curve showed that the RF model had a good net clinical benefit in clinical applications, as shown in Figure 2F. The above results indicated that the RF model had the best predictive performance among the 5 ML models.

**TABLE 2:** Performance of the 5 ML models on the internal validation set.

Models	AUC	Accuracy	Sensitivity	Specificity	PPV	NPV	F1 score
RF	0.863	0.795	0.776	0.861	0.757	0.825	0.765
XGBoost	0.852	0.786	0.831	0.795	0.753	0.813	0.787
LR	0.843	0.772	0.761	0.831	0.728	0.807	0.742
DT	0.796	0.751	0.750	0.773	0.715	0.779	0.730
GBDT	0.854	0.783	0.836	0.777	0.743	0.819	0.783

**FIGURE 3:**
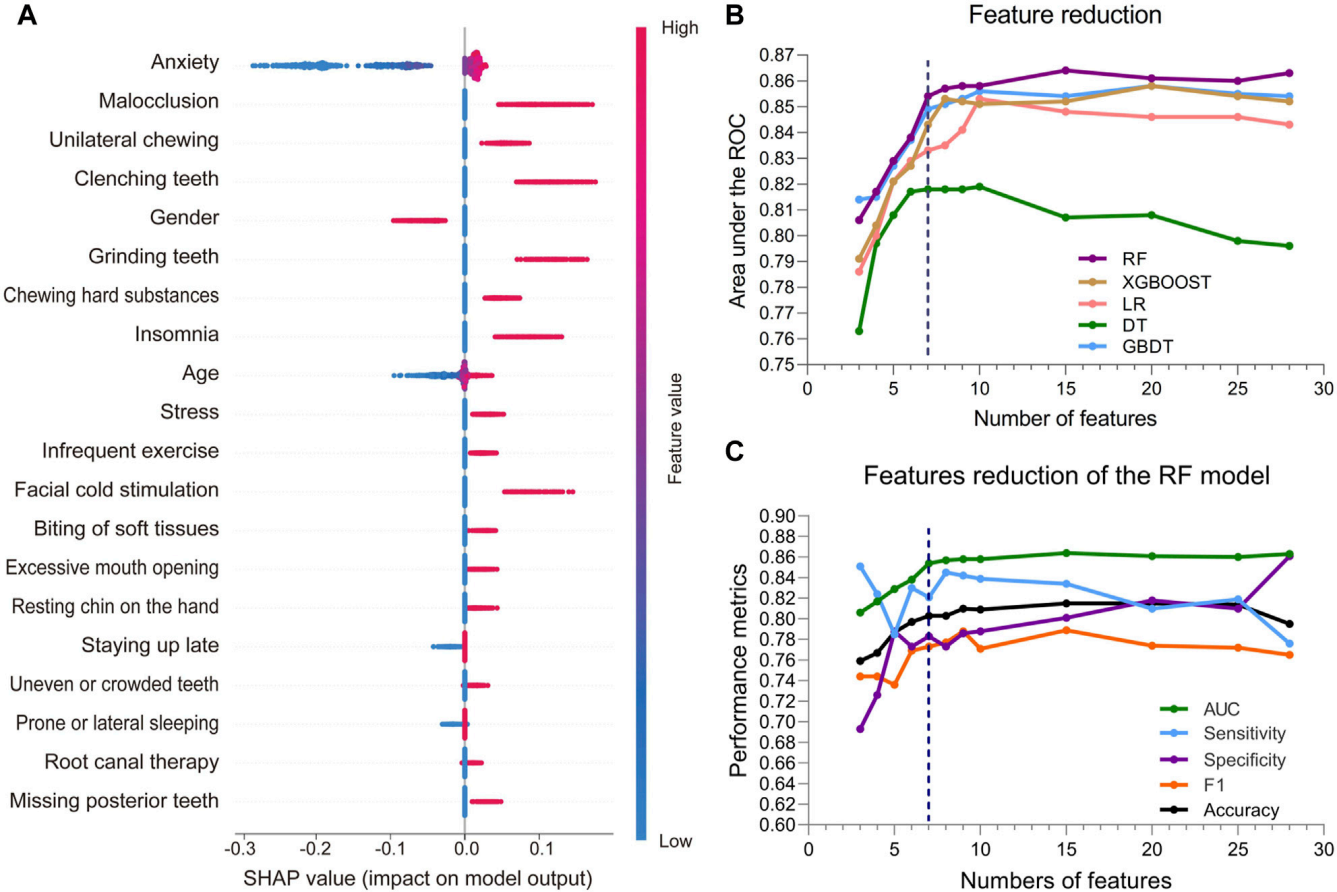
Feature attributes in SHAP for RF model and predictive performance with varied numbers of features. **(A)** The SHAP summary plot of the top 20 features for RF model. The horizontal coordinate represents the SHAP value, and each line indicates a feature. A dot represents the SHAP value of the corresponding feature for a patient, with high feature values shown in red and low feature values shown in blue. **(B)** AUCs of the five ML models with varied numbers of features. **(C)** AUC, sensitivity, specificity, and F1 score of the RF model with varied numbers of features.

The SHAP summary plot of the top 20 features for the RF model is shown in Figure 3A, while those for the other 4 ML models are shown in Supplementary Figure S2. These plots visualize the contribution of the different features to the model prediction results. Based on the feature importance ranking, we gradually eliminate unimportant features and reduce the number of features in the model from 28 to 3. During the feature reduction process, the RF model consistently retained the best predictive ability, as shown in Figure 3B. Therefore, we selected the RF models for the development of the final model.

### 3.3 Identification of the final model

**FIGURE 4:**
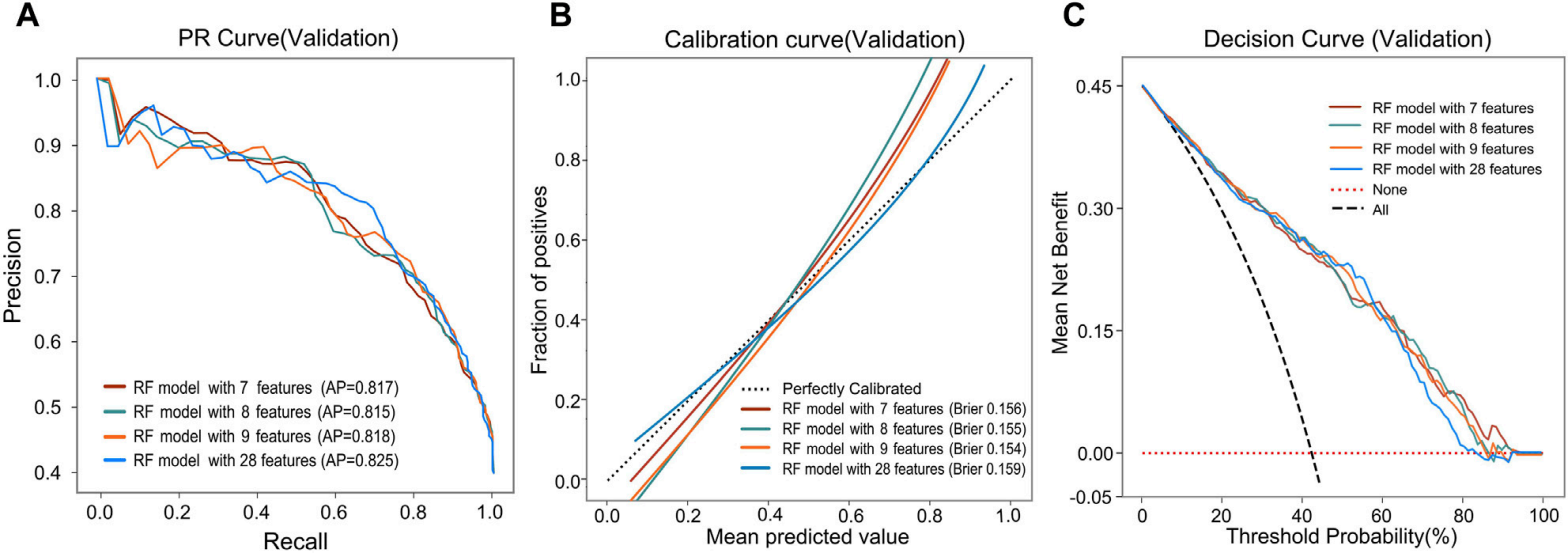
Predictive performance of the RF model after reducing features. **(A)** PR curves, **(B)** Calibration curves, and **(C)** DCA curves of the RF model with different features.

**FIGURE 5:**
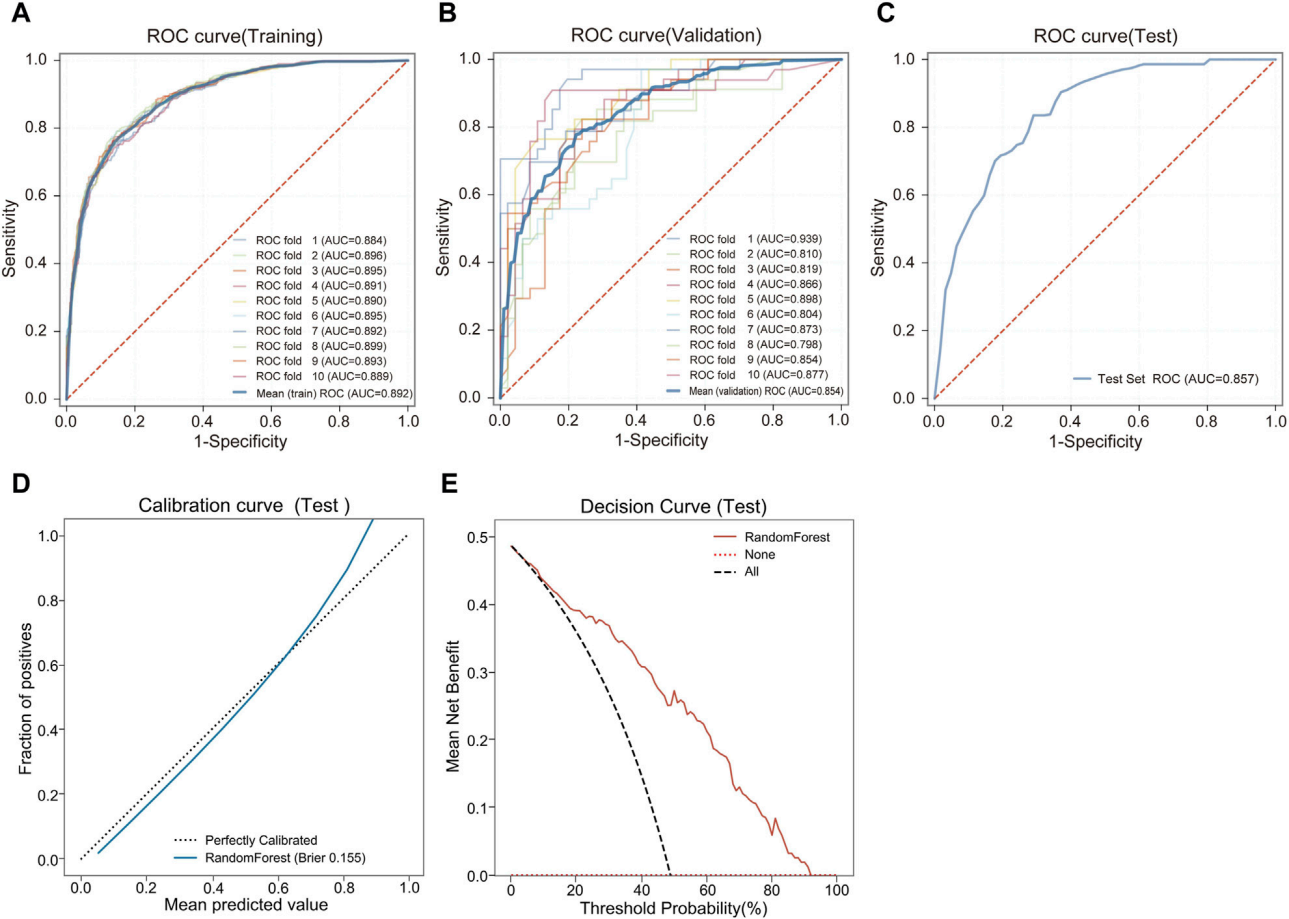
Predictive performance of the final RF model. **(A)** ROC and AUC of the training set. **(B)** ROC and AUC of the validation set. **(C)** ROC and AUC of the test set. **(D)** Calibration curve of the test set. **(E)** DCA curve of the test set.

During the feature reduction process of the RF model, we evaluated the effects of different numbers of features on the model performance, as shown in Figure 3C and Supplementary Table S3. When the number of features was increased from 6 to 7, the predictive ability of the model was significantly improved, with the AUC increasing from 0.838 to 0.854. However, further increases in the number of features did not result in a significant increase in predictive ability, and the model with 7 features performed similarly to the model with more features. Furthermore, the PR curve showed that the model with 7 features had a higher average accuracy (AP = 0.817), as shown in Figure 4A. Meanwhile, the calibration curve and the DCA curve showed well-calibrated and good net clinical benefit for models with 7 features, as shown in Figures 4B, C. Considering the model performance, complexity and computational efficiency, we selected the RF model with 7 features (gender, malocclusion, unilateral chewing, chewing hard substances, grinding teeth, clenching teeth, anxiety) as the final model. The mean AUC of the final model is 0.892 (95% CI, 0.869–0.916) on the training set and 0.854 (95% CI, 0.771–0.937) on the internal validation set, as shown in Figures 5A, B. In addition, the final model achieved an accuracy of 0.803 on the validation set, with a sensitivity of 0.821, specificity of 0.783, and F1 score of 0.773.

### 3.4 External test of the final model

The final model was externally tested using an independent dataset. The final RF model predicted TMD with an AUC of 0.857 (95% CI, 0.798–0.915), demonstrating the stability and reliability of the model, as shown in Figure 5C. The final model achieved an accuracy of 0.773 on the external test set, with a sensitivity of 0.712, specificity of 0.844, and F1 score of 0.750. The clinical performance of the model on an external test set was further evaluated. The calibration curve showed a good agreement between the predicted probabilities of the model and the actual observations. The Brier score of the model was 0.155, and further validation showed that the model had a good prediction accuracy, as shown in Figure 5D. The DCA curve showed that using our model to guide clinical decisions results in greater net benefit when the risk threshold is between 0.03 and 0.93, as shown in Figure 5E.

### 3.5 Model explanation

**FIGURE 6:**
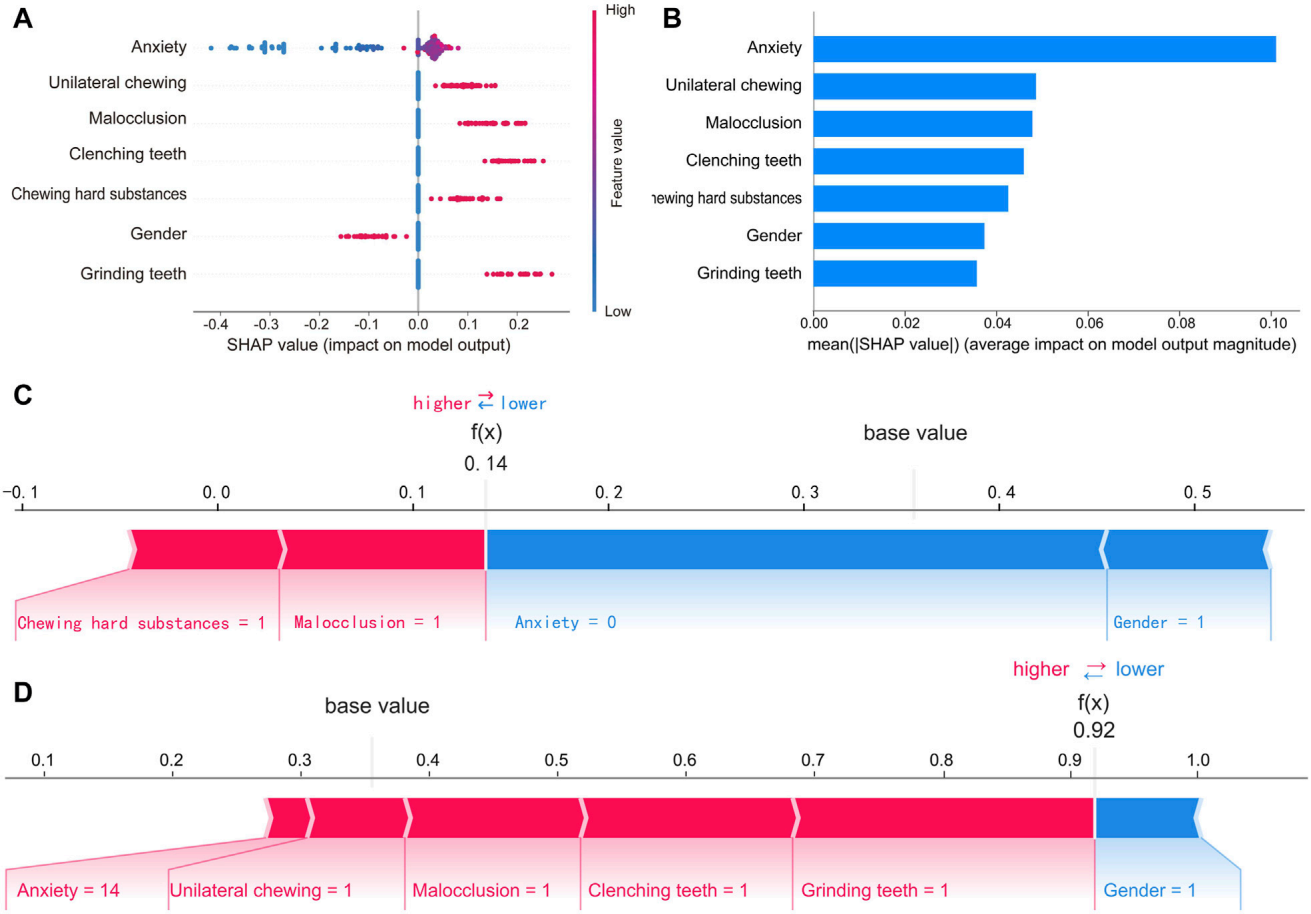
SHAP interprets of the final RF model. **(A)** Feature attributes in SHAP for the final model. The horizontal coordinate represents the SHAP value, and each line indicates a feature. A dot represents the SHAP value of the corresponding feature for a patient, with high feature values shown in red and low feature values shown in blue. **(B)** Ranking of feature importance in the final model. The matrix of the SHAP summary plot describes the importance of each feature of the final model. **(C)** SHAP force plot by patients without TMD and **(D)** with TMD. The features that increase the risk of TMD are shown in red, and those that decrease the risk of TMD are shown in blue. Predictors include unilateral chewing, malocclusion, clenching teeth, chewing hard substances, and grinding teeth, where 1 indicates the presence of the factor and 0 indicates its absence. Gender is coded as 0 for female and 1 for male. Anxiety level is measured by GAD-7 scores.

To make the prediction model more transparent, we used the SHAP algorithm to explain the prediction model. The SHAP algorithm quantified the contribution of each feature to the prediction result and provided an explanation for the final output of the model. Figure 6A shows the 7 features of the model and the specific contribution of each feature to the model output. Figure 6B shows the ranking of the importance of the 7 features as follows: anxiety, unilateral chewing, malocclusion, clenching teeth, chewing hard substances, gender, and grinding teeth. In addition, the interpretability of the model was demonstrated through two specific cases. One was a low-risk case with a low SHAP predictive score (0.14); the other was a high-risk case with a high SHAP predictive score (0.92), as shown in Figures 6C, D.

## 4 Discussion

In this study, using the ML approach, we identified the 7 important predictors of TMD occurrence in adults (anxiety, unilateral chewing, malocclusion, clenching teeth, chewing hard substances, gender, and grinding teeth), and based on these factors, we successfully developed an interpretable risk prediction model for TMD in adults, which provides new ways for TMD prevalence risk assessment and disease diagnosis.

The innovation of the study is its broad and deep consideration of multidimensional predictors. The study included 28 predictors covering multiple dimensions, including demographic information, oral-related medical history, occlusal factors, oral behavioral habits, lifestyle habits, sleep status, and psychological state. This multidimensional consideration allows the model to more comprehensively and accurately assess the risk of developing TMD. Furthermore, we used a feature reduction method to screen predictors, optimizing model performance by progressively eliminating less important predictive factors. Finally, based on the comprehensive consideration of model utility, efficiency and accuracy, we identified the 7 most important predictors for model construction. This approach not only improves the predictive performance and computational efficiency of the model, but also enhances the interpretability and stability of the model, making it more applicable to medical practice.

Another innovation of this study is the use of the SHAP algorithm to provide both global and local explanations for the model. The SHAP algorithm is based on the Shapley values from cooperative game theory. It calculates the marginal contribution of each feature across different combinations to explain the impact of each feature on the model’s prediction outcomes. By simulating the contribution of each feature to various model decisions, SHAP quantifies the importance of features on a unified scale. Due to its ability to provide consistent feature importance explanations across various machine learning models, SHAP has become widely used for visualizing and interpreting complex algorithms. We not only quantified the specific contributions of the 7 predictors to the model’s predicted outcomes, but also ranked them in order of importance based on these contributions, thus providing clinicians with more comprehensive and detailed reference information. In addition, the model can clearly show the impact of different predictors on the prediction results for each specific individual. This personalized explanation enables clinicians to understand each patient’s specific risk profile and formulate more precise and effective preventive measures and treatment strategies. The use of the SHAP algorithm significantly enhances clinicians’ understanding and application of the model, further improving the model’s practicality and reliability in clinical decision making.

The results of this study showed that gender is a significant risk factor for the occurrence of TMD in adults. Studies have indicated that the prevalence of TMD in females is almost twice that of males (Bueno et al., 2018). This gender disparity is primarily attributed to biological factors, hormonal fluctuations, and psychosocial factors. Estrogen and progesterone in females may play critical roles in regulating pain perception, inflammatory responses, and cartilage protection of the TMJ, and changes in these hormones may increase the risk of TMD in women (Ribeiro-Dasilva et al., 2009; Farook and Dudley, 2023). Additionally, structural characteristics of the TMJ in females, such as a shallow glenoid fossa and a larger condyle, may also increase the risk of joint instability (Ayyıldız et al., 2021).

The association between malocclusion and TMD has long been a subject of debate. Malocclusion alters the condylar movement trajectory, subjecting the TMJ to additional stress, and leading to injury of the articular disc and condylar cartilage, thereby precipitating TMD symptoms (Ono and Yonemitsu, 2024). Studies have shown a positive correlation between malocclusion complexity and the occurrence of TMD, with greater malocclusion complexity increasing the risk of TMD (Zúñiga-Herrera et al., 2023). Occlusal features such as deep overjet, ≥ 5 mm overbite, anterior open bite, and posterior crossbite are recognized as common manifestations of malocclusion in TMD (Macrì et al., 2022). Simulation studies by Usui et al. (2003) suggest that patients with anterior open bite may exert greater strain on the TMJ with their occlusal force. Thilander et al. (2002) observed a significant correlation between TMD and posterior crossbite, emphasizing the importance of early correction of malocclusion. A study by Henrikson (2000) confirmed that orthodontic treatment helps to improve the symptoms of TMD patients with the presence of malocclusion. However, some scholars suggest that existing evidence is insufficient to fully establish the relationship between occlusion factors and TMD, warranting further in-depth research (Manfredini et al., 2017; Al-Ani, 2020). Despite ongoing controversies regarding the role of occlusal factors as etiological agents in TMD, our findings supported the viewpoint of considering occlusal intervention when evaluating and treating TMD patients.

In terms of oral parafunctional movements, our study showed that teeth grinding and clenching are important risk factors for the occurrence of TMD. Teeth grinding and clenching increased the risk of TMD, consistent with previous research findings (Karakis and Dogan, 2015; Da Silva et al., 2017; Yalçın Yeler et al., 2017; Marpaung et al., 2018; Wu et al., 2021; Câmara-Souza et al., 2023). Prolonged grinding and clenching exert sustained pressure on the joints, potentially leading to disc displacement, capsular and ligamentous laxity, thereby precipitating symptoms such as pain, clicking, and limited mouth opening. Additionally, these behaviors may increase the tension of masticatory muscles and induce compensatory muscle hypertrophy, as evidenced by significantly increased masseter and temporalis muscle thickness in TMD patients compared to healthy individuals (Garip et al., 2018). In this study, unilateral chewing was also identified as a significant risk factor for TMD. Several studies have confirmed that individuals habitually engaging in unilateral chewing are more prone to TMD symptoms (Yalçın Yeler et al., 2017; Paulino et al., 2018; Wu et al., 2021). Unilateral chewing alters the normal movement path of the condyle, increasing the complexity of its movement trajectory, thereby increasing the risk of developing TMD. Moreover, Prolonged unilateral chewing can lead to internal force imbalances within the TMJ, potentially triggering structural remodeling of the joint and alterations in the fiber structure of masticatory muscles, consequently resulting in mandibular asymmetry and discordant bilateral muscle movements, and increasing the risk of developing TMD (Heikkinen et al., 2022). Our study also showed that long-term chewing of hard substances is another important risk factor for developing TMD. A study by Paulino et al. (2018)confirmed a significant correlation between the occurrence of TMD and chewing hard substances, and a study by Akhter et al. (2004) in Bangladeshi adolescents also found the frequency and type of hard food intake were significantly associated with the development of TMD. Prolonged chewing of hard substances increases the burden on the TMJ, leading to fatigue and spasms of the masticatory muscles, and may also lead to abnormal wear of the teeth, which affects the balance of masticatory forces and increases the risk of developing TMD.

This study further confirmed that psychological factors are closely related to the occurrence of TMD. Studies have shown that anxiety and depression are significantly associated with the occurrence of TMD (Paulino et al., 2018; Yap et al., 2022a; Natu et al., 2018; Al-Khotani et al., 2016; Castaño Joaqui et al., 2023; Simoen et al., 2020). Psychological disorders increase the risk of TMD by activating the stress response, inhibiting immune function, and promoting the development of chronic inflammation (Jo et al., 2016). These negative emotions can also cause muscle tension in the temporomandibular region, increasing the burden on the TMJ, which may trigger or exacerbate TMD symptoms. Studies have also shown that the association between psychological disorders and TMD may be due to behaviors triggered by psychological stress, such as bruxism and clenching, which significantly increase the risk of TMD (Karibe et al., 2015; Al-Khotani et al., 2016; de Paiva Bertoli et al., 2018). In the correlation analysis of predictive factors, we found Spearman’s correlation coefficient of 0.83 for anxiety and depression, indicating that they are highly correlated. Including them both in the model may lead to redundant effects and reduce the predictive power of the model. In clinical practice, the prevalence of anxiety is generally significantly higher than that of depression (Li et al., 2019). Moreover, anxiety symptoms are often easier to identify and manage, as patients tend to have stronger cognitive recognition and coping abilities for anxiety, which can usually be alleviated more quickly through interventions. In contrast, the treatment of depression tends to require a longer period, and the intervention strategies are more complex. Additionally, studies by Liou et al. (2023) and Medeiros et al. (2020)) suggest that anxiety may have a greater impact on the risk of TMD than depression. Based on clinical experience and research, we prioritized anxiety as a predictor to eliminate multicollinearity, improve model accuracy and stability, and better reflect actual clinical conditions.

In the detection and prediction of TMJ disorders, researchers have employed various methods. Fang et al. (2023) used the LASSO method to select important features and developed a logistic regression model for detecting degenerative TMJ disease based on cephalometric images. Bianchi et al. (2020) constructed a combination model of XGBoost and LightGBM for diagnosing TMJ osteoarthritis by selecting interaction variables. Vinayahalingam et al. (2023) achieved automatic TMJ image segmentation using a 3D U-Net deep learning model. Jp et al. (2023) used the Chi-squared Automatic Interaction Detector (CHAID) algorithm for variable selection and predicted the risk for TMD in adolescents.

In this study, we used 5 ML algorithms: RF, XGBoost, GBDT, DT, and LR to develop a risk prediction model for adult TMD. Random Forest mitigates overfitting and improves model stability and generalization by integrating multiple decision trees. XGBoost and GBDT, as boosting algorithms, balance bias and variance effectively. The DT, while simple and prone to overfitting, handles non-linear features well. LR offers interpretability and fast modeling, making it ideal for baseline comparisons. Ultimately, the RF model showed optimal performance in predicting adult TMD risk, offering both stability and generalizability, and providing strong support for clinical application. Compared to other studies, we employed the SHAP algorithm for predictor screening. By visualizing each factor’s contribution to the model’s predictions, we progressively removed less important predictors to achieve variable selection. After feature reduction, the model demonstrated strong generalization capabilities. An AUC of 0.854 and an accuracy of 0.803 for the validation set, and an AUC of 0.857 with an accuracy of 0.773 for the external test set. These results indicate that the model performs well not only during development but also maintains stable predictive performance in real-world clinical applications. Importantly, the data required for the model are easily accessible and suitable for collection in clinical and epidemiological studies. Since the model does not rely on imaging devices or biomarker testing, it offers high generalizability and practicality, making it well-suited for large-scale clinical implementation.

Although this study has made some progress in identifying TMD risk factors and developing a risk prediction model, it also has certain limitations. Firstly, the sample size and diversity of the study were limited, and future research should consider using larger and more diverse samples to improve the generalizability of the results. Secondly, because the pathogenesis of TMD has not been fully clarified, the model may not be able to comprehensively capture all factors that are closely associated with the risk of TMD. To obtain a more accurate and comprehensive TMD risk assessment, future studies need to further explore the pathogenesis of TMD in depth and identify more potential influencing factors to further improve the accuracy and usefulness of the prediction model.

## 5 Conclusion

We applied ML methods to successfully identify 7 important risk factors for the occurrence of TMD in adults and developed an efficient and interpretable TMD risk prediction model. This model not only demonstrates good predictive performance, but also further enhances its clinical applicability through SHAP methods. It will help clinicians to more accurately and conveniently predict and assess the risk of TMD in adults, and provide decision support for the implementation of personalized prevention and medical interventions.

## Data Availability

The raw data supporting the conclusions of this article are available from the corresponding author upon reasonable request.
