# Supplementary material for: Predicting temporomandibular disorders in adults using interpretable machine learning methods: a model development and validation study
Source: Front Bioeng Biotechnol. 2024 Nov 5;12:1459903. doi: 10.3389/fbioe.2024.1459903 (PMC11573567; doi:10.3389/fbioe.2024.1459903)
Supplement: Supplementary file 1 [file DataSheet1.pdf]

Supplementary Material

1 Supplementary Tables

Supplementary Table S1 Demographics and clinical characteristics on the external test cohort

Characteristic		Total	Non-TMD (n=77)	TMD (n=73)	p-value
Age, Mean ± SD		35.09 ± 11.51	36.77 ± 11.58	33.32 ± 11.25	0.066
Gender, n(%)	Female	92(61.33)	38(49.35)	54(73.97)	0.002
	Male	58(38.67)	39(50.65)	19(26.03)	
Orthodontics, n(%)	No	127(84.67)	70(90.91)	57(78.08)	0.029
	Yes	23(15.33)	7(9.09)	16(21.92)	
Root canal therapy, n(%)	No	104(69.33)	60(77.92)	44(60.27)	0.019
	Yes	46(30.67)	17(22.08)	29(39.73)	
Facial cold stimulation, n(%)	No	135(90.00)	74(96.10)	61(83.56)	0.010
	Yes	15(10.00)	3(3.90)	12(16.44)	
Unilateral chewing, n(%)	No	69(46.00)	45(58.44)	24(32.88)	0.002
	Yes	81(54.00)	32(41.59)	49(67.12)	
Chewing hard substances, n(%)	No	84(56.00)	54(70.13)	30(41.10)	<0.001
	Yes	66(44.00)	23(29.87)	43(58.90)	
Chewing gum, n(%)	No	131(87.33)	71(92.21)	60(82.19)	0.065
	Yes	19(12.67)	6(7.79)	13(17.81)	
Biting of soft tissues, n(%)	No	110(73.33)	63(81.82)	47(64.38)	0.016
	Yes	40(26.67)	14(18.18)	26(35.62)	
Grinding teeth, n(%)	No	123(82.00)	73(94.81)	50(68.49)	<0.001
	Yes	27(18.00)	4(5.19)	23(31.51)	
Clenching teeth, n(%)	No	112(74.67)	67(87.01)	45(61.64)	<0.001
	Yes	38(25.33)	10(12.99)	28(38.36)	
Excessive mouth opening, n(%)	No	100(66.67)	62(80.52)	38(52.05)	<0.001
	Yes	50(33.33)	15(19.48)	35(47.95)	
Mouth breathing, n(%)	No	91(60.67)	54(70.13)	37(50.68)	0.015
	Yes	59(39.33)	23(29.87)	36(49.32)	
Uneven o rcrowded teeth, n(%)	No	92(61.33)	55(71.43)	37(50.68)	0.009
	Yes	58(38.67)	22(28.57)	36(49.32)	
Missing posterior teeth, n(%)	No	140(93.33)	75(97.40)	65(89.04)	0.040
	Yes	10(6.67)	2(2.60)	8(10.96)	
Malocclusion, n(%)	No	101(67.33)	62(80.52)	39(53.42)	<0.001
	Yes	49(32.67)	15(19.48)	34(46.58)	
Faulty restoration, n(%)	No	126(84.00)	71(92.21)	55(75.34)	0.005
	Yes	24(16.00)	6(7.79)	18(24.66)	

Characteristic		Total	Non-TMD (n=77)	TMD (n=73)	p-value
Prone or lateral sleeping, n(%)	No	21(14.00)	16(20.78)	5(6.85)	0.014
	Yes	129(86.00)	61(79.22)	68(93.15)	
Infrequent exercise, n(%)	No	88(58.67)	60(77.92)	28(38.36)	<0.001
	Yes	62(41.33)	17(22.08)	45(61.64)	
Resting chin on the hand, n(%)	No	75(50.00)	49(63.64)	26(35.62)	<0.001
	Yes	75(50.00)	28(36.36)	47(64.38)	
Staying up late, n(%)	No	38(25.33)	27(35.07)	11(15.07)	0.005
	Yes	112(74.67)	50(64.93)	62(84.93)	
Prolonged mobile phone use, n(%)	No	19(12.667)	15(19.48)	4(5.48)	0.010
	Yes	131(87.33)	62(80.52)	69(94.52)	
Insomnia, n(%)	No	104(69.33)	64(83.12)	40(54.79)	<0.001
	Yes	46(30.67)	13(16.88)	33(45.21)	
Smoking, n(%)	No	118(78.67)	65(84.42)	53(72.60)	0.078
	Yes	32(21.33)	12(15.58)	20(27.40)	
Drinking, n(%)	No	128(85.33)	68(88.31)	60(82.19)	0.290
	Yes	22(14.67)	9(11.69)	13(17.81)	
Obesity, n(%)	No	124(82.67)	68(88.31)	56(76.71)	0.061
	Yes	26(17.33)	9(11.69)	17(23.29)	
Stress, n(%)	No	77(51.33)	51(66.23)	26(35.62)	<0.001
	Yes	73(48.67)	26(33.77)	47(64.38)	
Anxiety, median[IQR]		5.00[1.00,7.00]	2.00[0.00,6.00]	7.00[4.00,7.00]	<0.001
Depression, median[IQR]		5.00[1.00,8.00]	3.00[0.00,7.00]	8.00[4.00,9.00]	<0.001

Supplementary Table S2 Comparison of demographics and clinical characteristics on the development cohort and the external test cohort

Characteristic	Development cohort (n=799)	Test cohort (n = 150)	p-value
Age			0.582
Median (IQR)	36.06 ± 13.22	35.09 ± 11.51	
Gender, n(%)			0.385
Female	460(57.57)	92(61.33)	
Male	339(42.43)	58(38.67)	
Orthodontics, n(%)			0.985
No	676(84.61)	127(84.67)	
Yes	123(15.39)	23(15.33)	
Root canal therapy, n(%)			0.472
No	577(72.22)	104(69.33)	
Yes	222(27.78)	46(30.67)	
Facial cold stimulation, n(%)			0.886
No	716(89.61)	135(90.00)	
Yes	83(10.39)	15(10.00)	

Characteristic	Development cohort (n=799)	Test cohort (n = 150)	p-value
Unilateral chewing, n(%)			0.221
No	411(51.44)	69(46.00)	
Yes	388(48.56)	81(54.00)	
Chewing hard substances, n(%)			0.052
No	514(64.33)	84(56.00)	
Yes	285(35.67)	66(44.00)	
Chewing gum, n(%)			0.632
No	686(85.86)	131(87.33)	
Yes	113(14.14)	19(12.67)	
Biting of soft tissues, n(%)			0.951
No	584(73.09)	110(73.33)	
Yes	215(26.91)	40(26.67)	
Grinding teeth, n(%)			0.399
No	677(84.73)	123(82.00)	
Yes	122(15.27)	27(18.00)	
Clenching teeth, n(%)			0.587
No	613(76.72)	112(74.67)	
Yes	186(23.28)	38(25.33)	
Excessive mouth opening, n(%)			0.968
No	534(66.83)	100(66.67)	
Yes	265(33.17)	50(33.33)	
Mouth breathing, n(%)			0.375
No	515(64.46)	91(60.67)	
Yes	284(35.54)	59(39.33)	
Uneven or crowded teeth, n(%)			0.930
No	487(60.95)	92(61.33)	
Yes	312(39.05)	58(38.67)	
Missing posterior teeth, n(%)			0.397
No	729(91.24)	140(93.33)	
Yes	70(8.76)	10(6.67)	
Malocclusion, n(%)			0.334
No	505(63.20)	101(67.33)	
Yes	294(36.80)	49(32.67)	
Faulty restoration, n(%)			0.382
No	647(80.98)	126(84.00)	
Yes	152(19.02)	24(16.00)	
Lateral sleeping position, n(%)			0.097
No	158(19.77)	21(14.00)	
Yes	641(80.23)	129(86.00)	
Infrequent exercise, n(%)			0.781
No	459(57.45)	88(58.67)	
Yes	340(42.55)	62(41.33)	
Prone or lateral sleeping, n(%)			0.109
No	456(57.07)	75(50.00)	
Yes	343(42.93)	75(50.00)	
Staying up late, n(%)			0.086
No	259(32.42)	38(25.33)	
Yes	540(67.58)	112(74.67)	
Prolonged mobile phone use, n(%)			0.138

Characteristic	Development cohort (n=799)	Test cohort (n = 150)	p-value
No	234(29.29)	35(23.33)	
Yes	565(70.71)	115(76.67)	
Insomnia, n(%)			0.132
No	640(80.10)	112(74.67)	
Yes	159(19.90)	38(25.33)	
Smoking, n(%)			0.399
No	652(81.60)	118(78.67)	
Yes	147(18.40)	32(21.33)	
Drinking, n(%)			0.086
No	724(90.61)	129(86.00)	
Yes	75(9.39)	21(14.00)	
Obesity, n(%)			0.353
No	684(85.61)	124(82.67)	
Yes	115(14.39)	26(17.33)	
Stress, n(%)			0.184
No	457(57.20)	77(51.33)	
Yes	342(42.80)	73(48.67)	
Anxiety			0.053
Median[IQR]	4.00 [0.00, 7.00]	5.00 [1.00, 6.00]	
Depression			0.102
Median[IQR]	3.00 [0.00, 8.00]	5.00 [1.00, 7.00]	

Supplementary Table S3 Performance of the RF model with varied numbers of features for TMD prediction

Feature numbers	AUC	Accuracy	Sensitivity	Specificity	F1
3	0.806	0.759	0.851	0.693	0.744
4	0.817	0.767	0.824	0.726	0.744
5	0.829	0.787	0.785	0.788	0.736
6	0.838	0.797	0.83	0.773	0.769
7	0.854	0.803	0.821	0.783	0.773
8	0.857	0.803	0.845	0.773	0.777
9	0.858	0.810	0.842	0.786	0.788
10	0.858	0.809	0.839	0.788	0.771
15	0.864	0.815	0.834	0.801	0.789
20	0.861	0.815	0.810	0.818	0.774
25	0.860	0.814	0.819	0.810	0.772
28	0.863	0.795	0.776	0.861	0.765

The indexes represented the performance of the RF model with varied numbers of features in the internal validation set.

2 Supplementary Figures

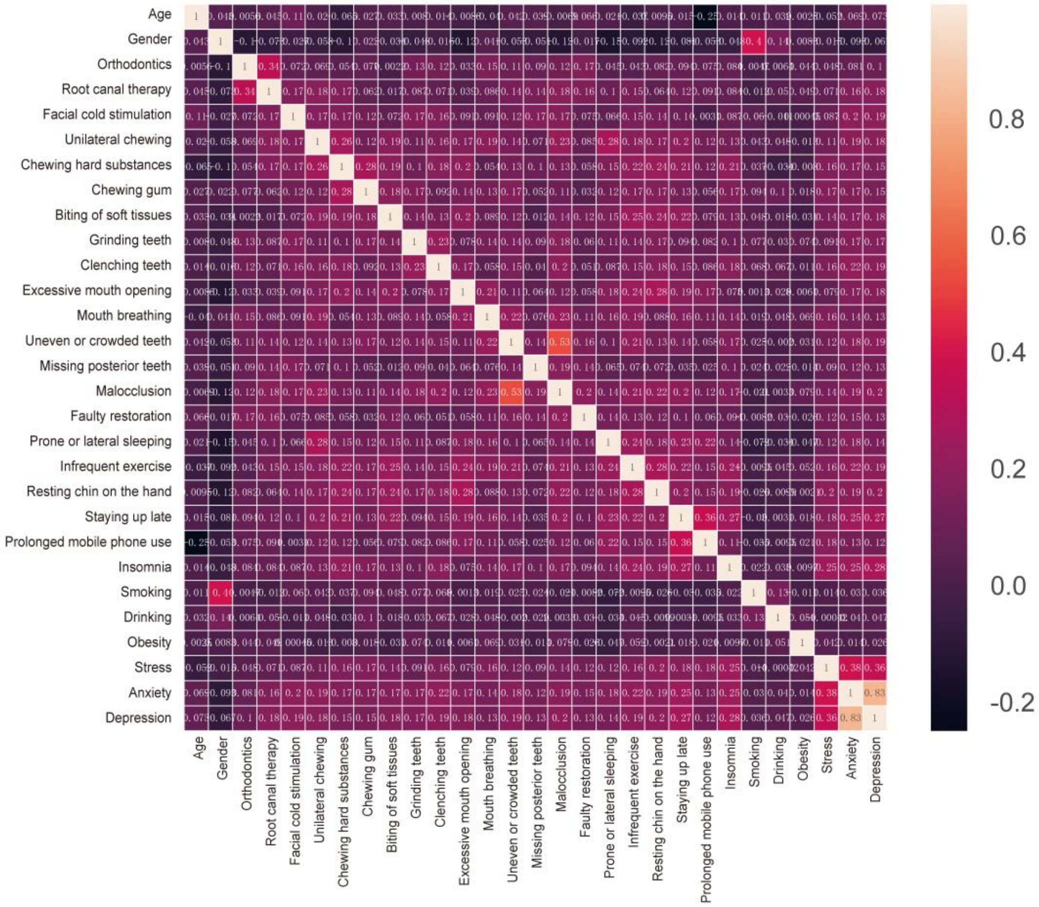

Supplementary Figure S1 Heat map of Spearman's correlation analyses among variables. Variables from the training set were included in Spearman's correlation analyses.

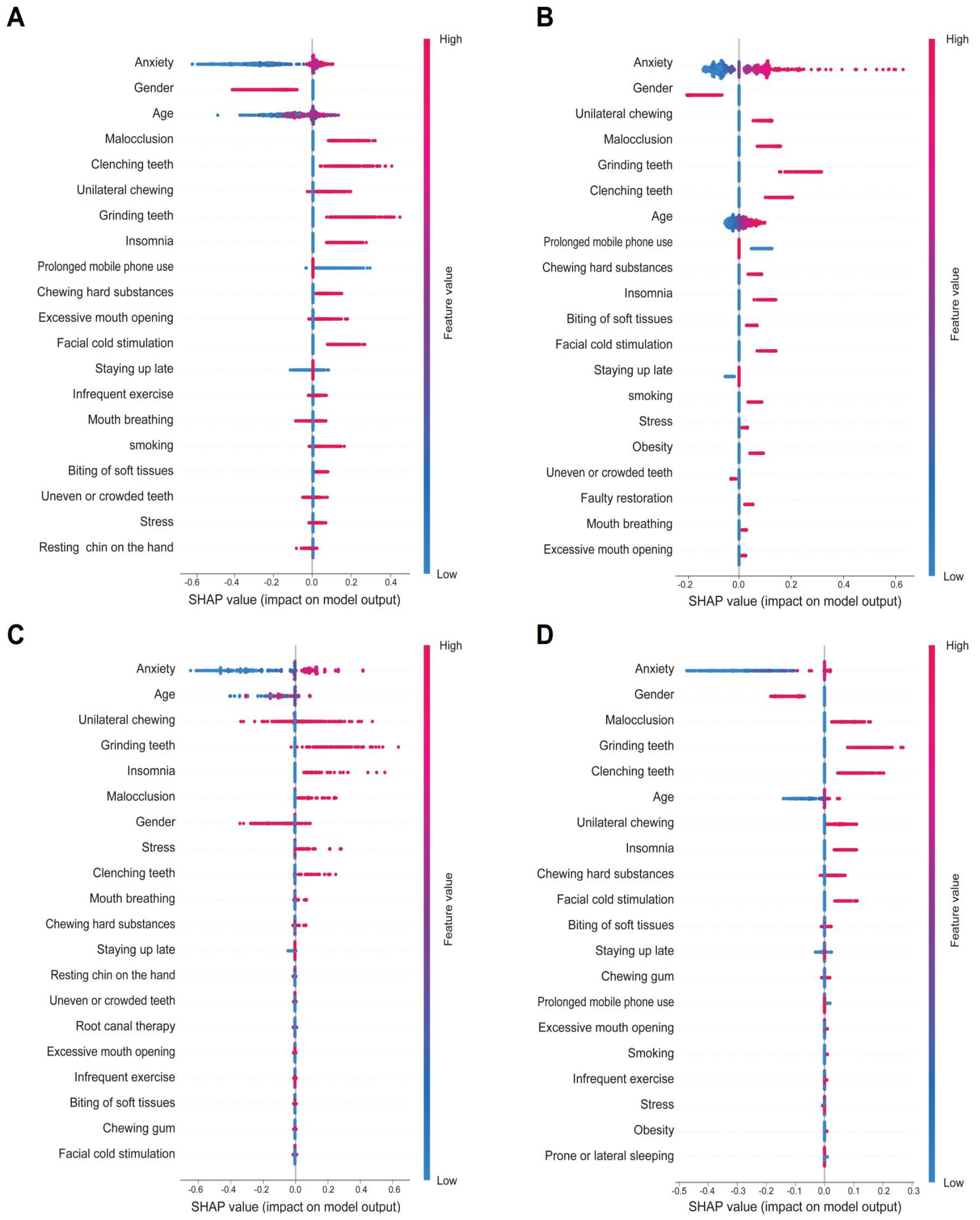

Supplementary Figure S2 The SHAP summary plot of the top 20 features for the four ML models. (A) XGboost. (B) LR. (C) DT. (D) GBDT.

3 DC/TMD Diagnostic Criteria

Diagnostic Criteria for the Most Common Temporomandibular Disorders: Symptom Questionnaire and Clinical Examination Items

All listed criteria in History (DC/TMD Symptom Questionnaire) and Examination (DC/TMD Examination Form) are required for the specific Diagnosis unless otherwise stated; each criterion is positive unless otherwise specified. Exact time period for time-relevant History or Examination items is not specified below since that is a function of how the items are constructed to suit a given purpose; the logic does not change according to time period. The time period as assessed by history items for disc displacement without reduction vs with reduction is relative.

Source: Schiffman E, Ohrbach R, Truelove E, Look J, Anderson G, Goulet J-P, List T, Svensson P, Gonzalez Y, Lobbezoo F, Michelotti A, Brooks S, Ceusters W, Drangsholt M, Ettlin D, Gaul C, Goldberg LJ, Haythornthwaite J, Hollender L, Jensen R, John MT, deLaat A, deLeeuw R, Maixner W, van der Meulen M, Murray GM, Nixdorf DR, Palla S, Petersson A, Pionchon P, Smith B, Visscher C, Zakrzewska J, and Dworkin SF (2014). Diagnostic Criteria for Temporomandibular Disorders (DC/TMD) for Clinical and Research Applications: Recommendations of the International RDC/TMD Consortium Network and Orofacial Pain Special Interest Group. *Journal of Oral & Facial Pain and Headache* 28:6-27.

Version 2/6/2020

	History		Examination	
Disorder	*Criteria*	*SQ*	*Criteria*	*Examination Form*
**Pain Disorders**				
Myalgia<br>(ICD-9 729.1)<br>• Sens 0.90<br>• Spec 0.95	Pain in a masticatory structure	SQ3	Confirmation of pain in masticatory muscle(s)	E1a
	Pain modified by jaw movement, function, or parafunction	SQ4	Familiar pain in masticatory muscle(s) with either muscle palpation or maximum opening	E4b, E4c, or E9: familiar pain in temporalis or masseter, or in other masticatory muscles if also relevant; **or**<br>E10: familiar pain in supplemental muscles, if E10 included
Myalgia Subtypes				
Local Myalgia<br>(ICD-9 729.1)<br><br>Sens and Spec not established	[same as for Myalgia]	[SQ3 & SQ4]	Confirmation of pain in masticatory muscle(s)	E1a
			Familiar pain with muscle palpation	E9: familiar pain in masseter or temporalis; **or**<br>E10: familiar pain in supplemental muscles, if E10 included
			Pain remains local to the area of stimulation	E9: negative referred and spreading pain; **and**<br>E10: negative referred and spreading pain, if E10 included
Myofascial Pain with Spreading<br>(ICD-9 729.1)<br><br>Sens and Spec not established	[same as for Myalgia]	[SQ3 & SQ4]	Confirmation of pain in masticatory muscle(s)	E1a
			Familiar pain with muscle palpation	E9: familiar pain in masseter or temporalis; **or**<br>E10: familiar pain in supplemental muscles, if E10 included
			Spreading (but not referred) pain with muscle palpation	E9: spreading pain; **or**<br>E10: spreading pain, if E10 included;<br>**AND**<br>E9: negative referred pain; **and**<br>E10: negative referred pain, if E10 included

<table>
<tr><th></th><th colspan="2">History</th><th colspan="2">Examination</th></tr>
<tr><th>Disorder</th><th>Criteria</th><th>SQ</th><th>Criteria</th><th>Examination Form</th></tr>
<tr><td rowspan="3"><u>Myofascial Pain with Referral</u>
(ICD-9 729.1)
• Sens 0.86
• Spec 0.98</td><td>[same as for Myalgia]</td><td>[SQ3 & SQ4]</td><td>Confirmation of pain in masticatory muscle(s)</td><td>E1a</td></tr>
<tr><td></td><td></td><td>Familiar pain with muscle palpation</td><td>E9: familiar pain in masseter or temporalis; or
E10: familiar pain in supplemental muscles, if E10 included</td></tr>
<tr><td></td><td></td><td>Referred pain with muscle palpation</td><td>E9: positive referred pain; or
E10: positive referred pain, if E10 included</td></tr>
<tr><td rowspan="2">Arthralgia
(ICD-9 524.62)
• Sens 0.89
• Spec 0.98</td><td>Pain in a masticatory structure</td><td>SQ3</td><td>Confirmation of pain in TMJ(s)</td><td>E1a</td></tr>
<tr><td>Pain modified by jaw movement, function, or parafunction</td><td>SQ4</td><td>Familiar pain with TMJ palpation or range of motion</td><td>E4b, E4c, E5a-c, or E9: familiar pain in TMJ</td></tr>
<tr><td rowspan="2">Headache Attributed to TMD
(ICD-9 339.89 [other specified headache syndrome], or
ICD-9 784.0 [headache])
• Sens 0.89
• Spec 0.87

Note that for a secondary headache diagnosis, a primary diagnosis of either myalgia or arthralgia is required.</td><td>Headache of any type in temporal region</td><td>SQ5</td><td>Confirmation of headache in temporalis muscle</td><td>E1b</td></tr>
<tr><td>Headache affected by jaw movement, function, or parafunction</td><td>SQ7</td><td>Report of familiar headache in temporalis area from either:
a. Palpation of the temporalis muscle or
b. Range of motion of jaw</td><td>E4b, E4c, E5a-c, or E9: familiar headache pain in the temporalis muscle</td></tr>
</table>

	History		Examination	
Disorder	*Criteria*	*SQ*	*Criteria*	*Examination Form*
**Joint Disorders**				
Disc Displacement with Reduction<br>(ICD-9 524.63)<br>• Sens 0.34<br>• Spec 0.92	Current TMJ noises by history, **OR**	SQ8	Click(s) with opening <u>and</u> closing, **OR**	E6: (open & close) click, **OR**
	Patient reports noise during the examination	E6 or E7: noise reported by patient	Both (a) click with opening or closing, **and**	E6: (open or close) click, **and**
			(b) click with lateral or protrusive movements	E7: (protrusive or lateral) click
Disc Displacement with Reduction, with Intermittent Locking<br>(ICD-9 524.63)<br>• Sens 0.38<br>• Spec 0.98	[same as disc displacement with reduction]	[same as DD with red]	[same as disc displacement with reduction]	[same as DD with red]
	Current intermittent locking with limited opening	SQ11=yes<br>SQ12=no	When disorder present in clinic: maneuver required to open mouth	E8 (optional)
Disc Displacement without Reduction, with Limited Opening<br>(ICD-9 524.63)<br>• Sens 0.80<br>• Spec 0.97	Current* TMJ lock with limited opening	SQ9	Passive stretch (maximum assisted opening) < 40mm	E4c < 40mm including vertical incisal overlap
	Limitation severe enough to interfere with ability to eat	SQ10		
Disc Displacement without Reduction, without Limited Opening<br>(ICD-9 524.63)<br>• Sens 0.54<br>• Spec 0.79	Prior* TMJ lock with limited opening	SQ9	Passive stretch (maximum assisted opening) ≥ 40mm	E4c ≥ 40mm including vertical incisal overlap
	Limitation severe enough to interfere with ability to eat	SQ10		
Degenerative Joint Disease<br>(ICD-9 715.18)<br>• Sens 0.55<br>• Spec 0.61	Current TMJ noises by history, **OR**	SQ8	Crepitus during jaw movement	E6 or E7: crepitus detected by examiner
	Patient reports noise during the examination	E6 or E7: noise reported by patient		
Subluxation<br>(ICD-9 830.0)<br>• Sens 0.98<br>• Spec 1.00	TMJ locking or catching in wide open jaw position	SQ13	When disorder present in clinic: maneuver required to close mouth	E8 (optional)
	Unable to close mouth without specific maneuver	SQ14		

* "Current" and "Prior" (as based on S9) for distinguishing, respectively, the "with limitation" vs "without limitation" variants of Disc Displacement without Reduction are interpreted based on change over time as determined by history and as confirmed by the clinical examination for jaw range of motion.
